# Age-Related Variations in Breast Cancer Complications: Insights from Physical Medicine and Rehabilitation Clinical Practice

**DOI:** 10.7759/cureus.59745

**Published:** 2024-05-06

**Authors:** Mafalda Cunha, Marco Silva, Vitor Sousa, Rui Vaz, Maria João Azevedo, Ana Zão

**Affiliations:** 1 Physical Medicine and Rehabilitation, Unidade Local de Saúde Alto Ave, Guimarães, PRT; 2 Physical Medicine and Rehabilitation, Unidade Local de Saúde de Santo António, Porto, PRT

**Keywords:** patient management, clinical improvement, delayed consultation, functional limitations, lymphedema, shoulder pain, rehabilitation, age-related variations, breast cancer complications

## Abstract

Background

Breast cancer patients often experience complications related to the disease or its treatment. With the rising average life expectancy, age is becoming less of a factor in treatment decisions for this condition. This study aims to evaluate differences in breast cancer complications among various age groups in patients referred to a hospital’s physical medicine and rehabilitation (PMR) department.

Methodology

A retrospective study was conducted among all breast cancer patients evaluated in a PMR department between November 2019 and March 2021. Data were collected from patients’ clinical files. SPSS® version 24 (IBM Corp., Armonk, NY, USA) was used for data analysis.

Results

We assessed 85 patients with a mean age of 56 years, finding that shoulder pain was the primary referral reason, reported by 85.9% of patients, followed by lymphedema in 32% of cases. Patients aged 56-65 years exhibited greater deficits in shoulder mobility compared to those between 66 and 75 years old, as well as greater functional limitations compared to other age groups. Most patients reported symptoms post-surgery, with an average delay of 24 months in PMR consultation. Despite this, nearly all patients (89.3%) reported clinical improvement following interventions.

Conclusions

We found that individuals in the 56-65-year age group were more prone to develop functional and shoulder mobility limitations. Despite delayed consultation, the majority of patients experienced clinical improvement, highlighting the intervention effectiveness of PMR interventions. These findings suggest that age alone may not be a determining factor in the reported breast cancer sequelae, implying the influence of other contributing factors in patient management. Further research is needed to elucidate the underlying mechanisms contributing to the diverse burden of disease sequelae observed across different age groups and to devise tailored interventions.

## Introduction

The incidence of breast cancer is steadily increasing, positioning it as the most prevalent cancer among women and the second leading cause of cancer-related deaths in the United States [1].

Breast cancer patients frequently experience deficits stemming from the disease itself or as a consequence of the therapies undergone [2,3]. These include musculoskeletal complications such as pain, cutaneous sensitization, lymphedema, restricted joint range of motion (ROM), decreased muscle strength in the ipsilateral upper limb, sensibility impairments, and osteopenia or osteoporosis. Additionally, they may face various psychosocial challenges such as depression, anxiety, fatigue, cognitive impairment, body image concerns, and issues with sexual function, among others [1].

With the aging population, the proportion of patients aged 70-84 years newly diagnosed with breast cancer is expected to rise from 24.3% to 34.8% by 2030 [4]. However, treatment of older patients with breast cancer presents complexities related to functional status assessment, comorbidities, life expectancy, and treatment tolerance [1]. With increasing life expectancy, age is becoming less of a determining factor in treatment decisions for breast cancer [5]. Despite these challenges, research suggests that older patients can benefit from similar treatments as younger cohorts, including chemotherapy, which has been shown to offer survival benefits even for those with comorbid conditions [1].

Physical rehabilitation emerges as a crucial component in the management of breast cancer patients, offering relief from pain, fatigue, and treatment-related symptoms while enhancing ROM, physical functioning, and quality of life throughout the recovery journey [6,7]. Pain education and exercise have been suggested as effective in reducing postoperative pain [8]. Despite compelling evidence supporting the effectiveness of early rehabilitation in addressing lymphedema and showing improved outcomes in pain levels, ROM, and functional disability scores [9,10], there is still reluctance among surgeons to refer patients to physical therapy [11].

To our knowledge, the impact of age on impairment, activity limitation, and participation restriction in these patients has not been evaluated to date.

This study aims to assess the main complications of breast cancer patients attending a hospital’s physical medicine and rehabilitation (PMR) department and investigate the potential differences between age groups in breast cancer complications in these patients.

## Materials and methods

Study design

This retrospective observational study was conducted in the PMR department of Hospital Senhora da Oliveira Guimarães. It examined all patients diagnosed with breast cancer who were referred to PMR consultations within a specified timeframe, from November 2019 to March 2021.

Outcomes and data collection

Data were obtained by accessing patients’ electronic medical records. Demographic information including age and profession was recorded, along with the reason for referral to the PMR consultation. Additionally, details regarding breast cancer treatments, time elapsed since surgery, and a comprehensive characterization of patient complaints related to pain, mobility, functionality, presence of lymphedema, and paresthesias were documented. During the clinical assessment, various parameters were evaluated, including ROM limitations, presence of lymphedema, adhesions and retractions, tenderness upon palpation, and sensory changes. Furthermore, investigations were conducted regarding the presence of shoulder pathologies before the diagnosis of breast cancer. Diagnostic complementary examinations performed were recorded, as well as treatments administered up to the data collection date. The use of elastic sleeves as part of the treatment regimen was also documented.

Statistical analysis

The collected data underwent descriptive statistical analysis to characterize the patient sample and identify the patterns of complaints and relevant clinical findings.

Spearman and Pearson tests were used to analyze the correlation between variables. For continuous variables between groups, the Mann-Whitney and t-test were used. When dealing with categorical variables, Fisher and chi-square tests were utilized when the assumption was not verified. The level of statistical significance was set at p-values <0.05. Data analysis was conducted using SPSS® version 24 (IBM Corp., Armonk, NY, USA).

Ethical considerations

The respect for the dignity and autonomy of participants was strictly adhered to throughout the study, with rigorous measures implemented to safeguard the privacy and confidentiality of participants’ data. Despite the retrospective nature of the research and the absence of funding, all data were anonymized whenever possible, and only essential information was collected and disclosed, in accordance with the ethical principles of the Declaration of Helsinki. The corresponding author had full access to all of the data in the study and had final responsibility for the decision to submit for publication.

## Results

The detailed study results are summarized in Table 1.

**Table 1:** Characteristics of the study population.

Variable	Overall (n = 85)
Age distribution (%)	
≤45	6
46–55	41.7
56–65	32.1
66–76	10.7
Over 75	9.6
Surgery type (%)	
Tumorectomy	57
Mastectomy	43
Lymph node evaluation (%)	
Sentinel node testing	63
Lymph node dissection	37
Oncological treatments (%)	
Radiotherapy	92
Hormonotherapy	86
Chemotherapy	77
Symptoms and complications (%)	
Shoulder pain	85.9
Limited shoulder mobility	73.5
Functional limitations	63.9
Paresthesias	21.1
Physical examination findings (%)	
Tenderness	77.4
Myotendinous or cicatricial retractions	71.4
Lymphedema	26.4

A total of 85 breast cancer patients, with a mean age of 56 years (range = 30-88 years), were enrolled in this retrospective study. The distribution of age groups was as follows: 6% were 45 years old or younger, 41.7% were between 46 and 55 years old, 32.1% were between 56 and 65 years old, 10.7% were between 66 and 75 years old, and 9.6% were over 75 years old. Among the patients, 57% underwent tumorectomy while 43% underwent mastectomy. Sentinel lymph node testing was performed in 63% of cases, whereas 37% of patients underwent lymph node dissection. Most patients received radiotherapy (92%), hormonotherapy (86%), and/or chemotherapy (77%).

The primary reason for referral was shoulder pain, reported by 85.9% of patients, predominantly localized on the ipsilateral side of the breast disease (70%), with a smaller percentage experiencing contralateral (10%) or bilateral (20%) involvement. Additionally, 73.5% of patients reported limited shoulder mobility, 63.9% experienced functional limitations related to shoulder morbidity, and 21.1% reported paresthesias.

Symptoms were atypically manifested postoperatively, with 60% of patients reporting symptom onset during this period. The average delay between symptom onset and consultation was 24 months.

Upon examination, 77.4% of patients exhibited tenderness upon palpation of periarticular tissues, 71.4% presented with myotendinous or cicatricial retractions, and 26.4% presented with lymphedema. Lymphedema was identified as the primary complaint in 32% of patients and was significantly associated with greater limitations in shoulder ROM (p < 0.001). Patients who underwent lymph node dissection showed more pronounced joint mobility limitations compared to those who underwent sentinel node biopsy (p = 0.030).

When stratified by age, patients between 56 and 65 years old reported greater deficits in shoulder mobility (88.9%) compared to those between 66 and 75 years old (50%) (p = 0.033). Similarly, objective examination revealed a decreased joint ROM in 88.9% of patients aged 56-65 years compared to 33.3% of those aged 66-75 years (p = 0.003). Functional limitations were more prevalent in patients aged 56-65 years (85.2%) compared to those aged 66-75 years (27.5%) (p = 0.015) and those aged 46-55 years (54.3%) (p = 0.014).

No statistically significant differences were observed between age groups in other variables studied, including oncological treatments.

All patients received instruction in an exercise program and care practices. Of these, 57.5% underwent a rehabilitation program, of whom 89.3% reported clinical improvement.

## Discussion

A substantial proportion of breast cancer survivors experience upper body morbidity post-treatment, including pain, tightness, numbness, lymphedema, and limited ROM, among others [12].

In the study, participants were women diagnosed with breast cancer, averaging 56 years of age, all of whom underwent surgery and received various treatments such as radiotherapy, hormone therapy, and/or chemotherapy.

Shoulder pain was the leading cause of referral, followed by lymphedema in 32% of patients, consistent with findings from larger studies indicating that between 10% and 64% of women develop upper body symptoms post-breast cancer treatment, and approximately 20% experience lymphedema [12].

Regarding shoulder pain, it was predominantly observed on the ipsilateral side of the breast disease, aligning with previous findings [12,13]. However, a significant proportion of patients also reported contralateral or bilateral pain, which has been documented in other studies, potentially attributed to shoulder girdle misalignment and alterations in scapular kinematics [14]. Moreover, it is important to note that shoulder pain is a prevalent condition in the general population, carrying a significant burden of disease [15]. We also hypothesize that patients may compensate for the fear of damage and impairment in the ipsilateral shoulder by overloading the contralateral side, thereby increasing stress on the unaffected shoulder.

Importantly, lymphedema was correlated with decreased shoulder ROM when compared to women without lymphedema, consistent with findings from recent studies [16]. Additionally, patients who underwent lymph node dissection showed greater limitations in joint mobility than those who underwent sentinel node biopsy, confirming our expectations [12].

In our study, we observed that patients aged between 56 and 65 years exhibited greater limitations in shoulder mobility compared to the older age group. Additionally, they displayed more pronounced functional limitations compared to both younger and older age groups. To our knowledge, specific literature addressing this phenomenon is lacking. We hypothesize that individuals in the 56-65-year age group seem to be more prone to develop functional and shoulder mobility limitations due to various factors. These factors may include a higher likelihood of pre-existing shoulder pathology and decreased physical conditioning compared to younger individuals. Additionally, their potentially more active and demanding lifestyles could elevate the risk of musculoskeletal complications post-treatment, coupled with a potentially heightened inflammatory response and scarring following breast cancer treatment compared to older individuals. These differences may also be influenced by age-related biological and hormonal factors, as well as variations in treatment response across different age cohorts. However, further research is warranted to comprehensively elucidate the underlying mechanisms driving these observed differences and to devise age-specific treatment and rehabilitation approaches.

As anticipated, symptoms typically manifested postoperatively. However, it is noteworthy that there was an average delay of 24 months between symptom onset and consultation with the PMR department. This delay is concerning, especially considering that early rehabilitation is recognized as the standard clinical practice [3,9,10,17-19]. It underscores the need to improve the referral pathway within our hospital to ensure timely access to rehabilitation services. Despite this delay, it is encouraging to observe that almost all patients (89.3%) reported experiencing clinical improvement, highlighting the effectiveness of the interventions provided.

While our study offers important insights into breast cancer patients’ characteristics and clinical findings during PMR consultation, there are limitations to consider. The retrospective design may introduce biases from incomplete medical records. Reliance on electronic records may also limit data scope and accuracy. The small sample size from a single institution might not fully represent the broader population. These limitations should be noted when interpreting and generalizing the findings.

Further research is warranted to explore the underlying mechanisms driving the observed differences in shoulder mobility and functionality across different age groups, encompassing biological, hormonal, and treatment response factors. Additionally, exploring targeted interventions aimed at enhancing awareness and facilitating early access to rehabilitation services may mitigate delays in consultation and enhance functional outcomes for breast cancer patients.

## Conclusions

Our study sheds light on the prevalence of shoulder pain and lymphedema among breast cancer patients post-treatment, highlighting the significant burden these symptoms pose. We observed that patients aged between 56 and 65 years exhibited greater limitations in shoulder mobility and functional abilities, suggesting potential age-related factors contributing to these outcomes. Despite the delay in seeking consultation, the majority of patients reported clinical improvement, underscoring the effectiveness of the interventions provided. Further research is needed to better understand the underlying mechanisms and develop targeted interventions for improving outcomes in this population.
